# Clinical effects of pulmonary surfactant in combination with nasal continuous positive airway pressure therapy on neonatal respiratory distress syndrome

**DOI:** 10.12669/pjms.333.12227

**Published:** 2017

**Authors:** Congmin Zhang, Xiaojing Zhu

**Affiliations:** 1 Congmin Zhang, First Center Hospital of Baoding, Baoding 071000, Hebei Province, P. R. China; 2 Xiaojing Zhu, First Center Hospital of Baoding, Baoding 071000, Hebei Province, P. R. China

**Keywords:** Neonate, Pulmonary surfactant, Respiratory distress syndrome

## Abstract

**Objective:**

To analyze the therapeutic effect of pulmonary surfactant (PS) in combination with nasal continuous positive airway pressure (NCPAP) therapy on neonatal respiratory distress syndrome (NRDS).

**Methodology:**

Forty-nine neonates who were diagnosed as NRDS and admitted in our hospital from May 2014 to June 2015 were selected and divided into an observation group and a control group. The observation group was treated with PS and NCPAP. The control group was treated only with NCPAP. The clinical symptoms, pulmonary X-ray, arterial partial pressure of oxygen (PaO_2_) and prognosis of the two groups were observed.

**Results:**

Twelve hours after treatment, the partial pressure of carbon dioxide and oxygenation index decreased significantly (P<0.05), and PaO_2_ and ratio of arterial/pulmonary oxygen partial pressures increased significantly (P<0.05). Pulmonary X-ray examination showed that 78.3% of the observation group and 53.8% of the control group were relieved 12-24 hour after treatment, between which the difference was statistically significant (P<0.05). The improvement rate of the observation group was significantly higher than that of the control group (82.6% vs. 57.7%, P<0.05), the incidence of complications was significantly lower in the observation group (P<0.05), and the average length of stay in the observation group was significantly shorter (P<0.05).

**Conclusion:**

Both methods effectively treated NRDS, but PS in combination with NCPAP better improved oxygenation, reduced mortality and incidence of complications.

## INTRODUCTION

Neonatal respiratory distress syndrome (NRDS), also known as neonatal hyaline membrane disease, mainly threatens premature neonates with the gestational ages of <35 weeks. It is induced by the lack of pulmonary surfactant (PS),^1^,^2^ accounting for 50%-70% of premature deaths.^3^ Its main pathogenesis is alveolar collapse due to immature pulmonary development of neonates and insufficient PS secretion, and its clinical manifestations include progressive dyspnea and respiratory failure shortly after birth. PS is a natural mixture derived from porcine lung or bovine pulmonary homogenate with phospholipids and specific proteins as the main ingredients, containing 41% to 48% of lecithin and 51% to 58% of other phospholipids and 1% of hydrophobin. PS is capable of rapidly increasing pulmonary tidal volume, enhancing pulmonary compliance, reducing alveolar surface tension, maintaining alveolar stability, preventing alveolar collapse, reducing alveolar fluid leakage and improving alveolar defense.^4^,^5^ The combination of PS with nasal continuous positive airway pressure (NCPAP) can maintain the alveoli in an open state to fully improve the oxygenation status and reduce the respiratory work done by human body.^6^,^7^

NRDS is prone to being complicated by infection, pulmonary hemorrhage, intracranial hemorrhage, chronic pulmonary disease, patent ductus arteriosus, retinopathy, etc. At present, NRDS is mainly treated by NCPAP, mechanical ventilation and PS replacement therapy.^8^,^9^ However, mechanical ventilation is invasive, easily causing ventilator-related complications and posing high technical requirements. Our hospital began to combine PS with NCPAP for the treatment of NRDS since March 2014, obtaining satisfactory outcomes as follows.

## METHODS

### Baseline clinical data

Forty-nine neonates who were diagnosed as NRDS and admitted in our hospital from May 2014 to June 2015 were selected. This study was approved by the ethics committee of our hospital, and written informed consent was obtained from all guardians. NRDS was diagnosed referring to the fourth edition of Practical Neonatology.^1^ The neonates were divided into an observation group and a control group based on whether their guardians agreed to use PS. There were 23 cases in the observation group, including 13 boys and 10 girls, with the gestational ages of 27-36 weeks, (32.4 ± 1.6) on average. Their birth weights were 1.33-2.42 kg, (1.79 ± 0.45) on average, and the hospitalization ages were 16 min-9 hour. There were 26 cases in the control group, including 12 boys and 14 girls, with the gestational ages of 28-35 weeks, (31.5 ± 1.8) on average. Their birth weights were 0.95-3.10 kg, (1.83 ± 0.57) on average, and the hospitalization ages were 17 min-11 hour. There were no statistically significant differences in gender ratio, gestational age and birth weight between the two groups (P>0.05).

### Methods

The two groups NCPAP therapy after admission, with the oxygen concentration of 21%-80%, the gas flow of 6-8 L/min and the pressure of 4-7 cmH_2_O. Meanwhile, they were given warm keeping, nutritional support, infection prevention, fluid infusion, respiration-strengthening management and symptomatic treatment. The therapy was terminated when CPAP reduced to 2-3 cmH_2_O, the oxygen concentration reduced to 25%, and dyspnea was significantly alleviated or disappeared. If the oxygen concentration was >80%, pressure >6-7 cmH_2_O, and the oxygen saturation was still <85% after 6-8 hour of treatment, or type II respiratory failure appeared, the therapy was replaced with mechanical ventilation. For the observation group, exogenous PS (Calsurf, Beijing Double-Crane Pharmaceutical Co., Ltd., H20052128) was injected intratracheally as early as possible on the basis of the above-mentioned treatment.

### Administration methods

The neonates were administered with an improved method as early as possible. During traditional administration, the neonate was in the supine position. The trachea was catheterized and ventilated by a balloon. Then a small suction catheter was placed in the trachea catheter, through which drug was injected in 2-3 portions. Ventilation for oxygen supply was stopped in each injection. As to the improved method, sterilization was performed at 3-5 cm of the catheter outside the lips after tracheal intubation. Afterwards, the sterilized site was directly pierced with a No. 4-5 disposable scalp needle pierced in a degree of 30-40°, which was inserted 1-2 cm further along the horizontal direction of the catheter. Then drug (70-100 mg/kg each time) was slowly pushed using a syringe connected with the scalp needle and injected evenly. After injection, ventilation using balloon was continued for 3-5 minutes and then the catheter was removed to continue NCPAP therapy, during which ventilation for oxygen supply was not interrupted. Attention should be paid to the observation of vital signs during administration, and aspiration of sputum was forbidden within 6 hour under non-emergency conditions.

### Observation indices

The improvement of dyspnea and incidence of complications were observed before and after using PS respectively. The improvement rate, rate of changing into mechanical ventilation, as well as hospitalization time and cost of the two groups were compared.

In addition, pulmonary X-ray, arterial oxygen partial pressure (PaO_2_), oxyhemoglobin saturation, carbon dioxide partial pressure (PaCO_2_), oxygenation index (OI), and ratio of arterial/pulmonary oxygen partial pressures (a/AO_2_) were also compared.

### Statistical analysis

All data were analyzed by SPSS 17.0. The categorical data were expressed as (Ax±s). Inter-group means were compared by the t test. The numerical data were compared by the χ^2^ test. P<0.05 was considered statistically significant.

## RESULTS

### Main indices before and after treatment

Before treatment, the two groups had similar PaO_2_, PaCO_2_, a/AO_2_ and OI (P>0.05). After 12 hour of treatment, PaCO_2_ and OI significantly decreased, while PaO_2_ and a/AO_2_ significantly increased compared with those before treatment (P<0.01). Besides, a/AO_2_ values of the two groups were also significantly different after treatment (t=5.63, P<0.01) (Table-I).

**Table-I:** Main indices before and after treatment (x̄±s).

	*PaO_2_ (mmHg)*		*PaCO_2_ (mmHg)*		*a/AO_2_*		*OI*	

	*Observation*	*Control*	*Observation*	*Control*	*Observation*	*Control*	*Observation*	*Control*
Before	44.83±5.26	45.38±5.8	59.35±6.96	58.12±7.69	0.19±0.13	0.18±0.12	13.7±2.8	14.0±3.1
12 h after	80.75±11.73	75.69±10.85	49.32±6.35	49.96±7.20	0.68±0.17	0.37±0.17	6.9±1.9	9.8±3.0
T	13.13	11.26	5.23	18.31	11.03	4.89	9.25	5.10
P	<0.01	<0.01	<0.01	<0.01	<0.01	<0.01	<0.01	<0.01

### Clinical data before and after treatment

The clinical outcomes are listed in Table-II. The observation group had significantly higher improvement rate (P<0.05) and significantly lower incidence of complications (P<0.05) than those of the control group, but the two groups had similar rates of changing into mechanical ventilation (P>0.05).

**Table-II:** Clinical outcomes.

*Group*	*n*	*Improvement rate*	*Incidence of complications*	*Rate of changing into mechanical ventilation*
Observation	23	19 (82.6)	9 (39.1)	3 (14.3)
Control	26	15 (57.7)	18 (69.2)	5 (19.2)
χ^2^		4.12	4.98	0.04
P		<0.05	<0.05	<0.05

### Hospitalization time and cost of living neonates

The observation group had significantly shorter hospitalization time than that of the control group (P<0.01), but their hospitalization costs were similar (P>0.05) (Table-III).

**Table-III:** Hospitalization time and cost of living neonates (x̄±s).

*Group*	*n*	*Hospitalization cost (10,000 CNY)*	*Hospitalization time (day)*
Observation	19	1.89±0.49	14.6±4.1
Control	16	2.03±0.59	19.9±4.9
t		0.79	4.37
P		>0.05	<0.01

### Pulmonary X-ray results 12-24 h after treatment

**Fig.1:**
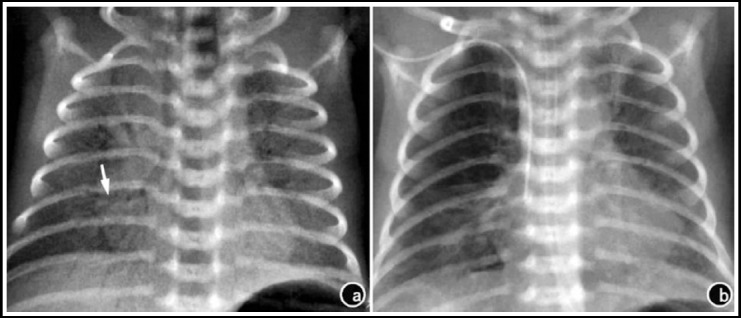
Pulmonary X-ray results of neonate No. 1 before and after treatment. a) 3 h after birth, before treatment; b) 30 h after PS treatment.

**Fig.2:**
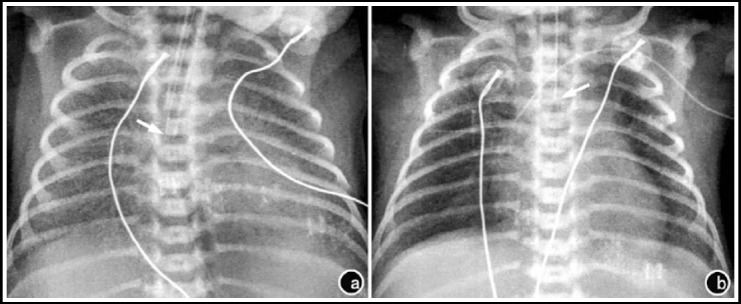
Pulmonary X-ray results of neonate No. 2 before and after treatment. a) 50 minutes after birth, before treatment; b) 8 hour after PS treatment.

As suggested by pulmonary X-ray examination 12-24 h after treatment, 78.3% of the observation group (18/23) was relieved, whereas only 53.8% of the control group (14/26) was alleviated, with a statistically significant difference (χ^2^=4.59, P<0.05) (Fig.1 and Fig. 2).

## DISCUSSION

NRD, a common severe disease in the neonatal period, is one of the important causes for premature death. The survivors are prone to suffering from chronic pulmonary diseases, seriously affecting their quality of life.^1^ As the treatment methods and technology continues to develop, the therapeutic effects on NRDS have been greatly improved. Oxygen therapy is one of the important measures to treat NRDS successfully.

NCPAP is a noninvasive ventilation method developed since the 1970s. It can stabilize chest wall activity and alleviate pleural dyssynergia in premature neonates, which has been widely used in the early treatment of NRDS.^10^,^11^ In addition, NCPAP provides controllable oxygen concentration and airway pressure, which can reduce the incidence of chronic pulmonary diseases and mechanical ventilation.^12^,^13^ In this study, PaCO_2_ and OI were decreased significantly, and PaO_2_ and a/AO_2_ were increased significantly 12 hour after treatment, which had significant differences between the two groups (P<0.05).

Dargaville et al. reported that Calsurf treatment of NRDS, which was safe and effective, could improve oxygenation and ventilation, and both the results of chest X-ray film and a/AO_2_ were significantly improved 8-24 hour after administration.^14^ a/AO_2_, which can be used as an indicator for RDS diagnosis and treatment, can reflect the anoxic status under oxygen uptake conditions, the ventilation function and the pulmonary vascular bed and alveolar damage, which is associated with good pulmonary blood flow but has no correlation with extrapulmonary organ failure.^15^,^16^ In this study, there were significant differences in OI and a/AO_2_ between the two groups 12 hour after treatment (P<0.01), and the difference of a/AO_2_ also had statistical significance (P<0.01). The difference in the review of chest X-ray film was significant between the two groups 12-24 hour after treatment (P<0.05). The improvement rate of the observation group was significantly higher than that of the control group (P<0.05), the incidence of complications was significantly lower in the observation group than in the control group (P<0.05), and the average length of stay in the observation group was significantly less than the control group (P<0.05), between which the differences were statistically significant, but the difference in the costs of hospitalization was not statistically significant (P>0.05). PS combined with NCPAP in the NRDS treatment not only improved the oxygenation and ventilation in NRDS neonates in a quick and effective way, but also prevented the progression of pulmonary diseases, reduced complications, shortened hospital stay, reduced NRDS mortality and improved premature prognosis and quality of life, without significantly increasing the costs of treatment. Therefore, PS combined NCPAP can be used as a priority treatment of NRDS.^17^

In this study, the observation group which used PS stressed administration as early as possible, and adopted the improved method of administration. Zhao et al. reported^18^ that the therapeutic effect of early administration (12 hours or less) was better than that of administration 12 hours later, and the treatment had a better effect if PS was given within six hour. As neonates suffer from respiratory distress, some protein substances may be released from alveoli, which can inhibit the activity of PS,^19^,^20^ affecting the therapeutic effect.

## CONCLUSION

In the traditional method of administration, drug was injected through the small catheter placed in the tracheal catheter, causing the separation of the resuscitator from the tracheal catheter. As a result, drug may be easily ejected during administration, resulting in drug waste and affecting the efficacy. Meanwhile, oxygen supply may be interrupted during operation. Hence, spO_2_ decline in neonates may aggravate the disease. In contrast, the improved method of administration is a closed operation process, which allows drug to be injected slowly and evenly, without waste or oxygen supply interruption. This strategy is thus worthy of clinical application.

### Authors’ Contributions

**CZ** designed this study and prepared this manuscript.

**CZ & XZ** performed this study.

**XZ** collected and analyzed clinical data.
